# Event and Comment

**Published:** 1910-03-15

**Authors:** 


### DENTAL REGISTER.

Vol. LXIV. March 15, 1910. No. 3.

### EVENT AND COMMENT.

PROSTHODONTIA. The idea prevails in some quarters that the artificial denture of former times is destired to be required less in the future than at present; largely because of the great progress we have made in utilizing roots of teeth formerly considered valueless, and because of the modern preventive methods of practice which seem to be just now claiming the attention of the profession. We should, however, not fail to note that artificial teeth are being manufactured in larger quantities today than ever before in the worlds history. Possibly many of these are exported to countries where the conservative and operative advance has not been so marked as in this country. The conservative dentist believes artificial dentures are going out of style, because he is not so often called upon to make them, which may be because his practice is of such a grade as to require little of this kind of work; but he should not forget that there are many practices in which the filling of a tooth is as rare an event as a denture is in his. Then again we have the commercial laboratories which are fast taking over the prosthetic work of even our best offices. A gentleman connected with a dental supply house which conducts a laboratory, recently told us that they could not get men enough to keep up with the demand made by the profession upon them. He said they employed six expert workmen and would be glad to get three or four more at once; they were adding every convenience to the laboratory that would expedite the work and working over time and were still unable to get out their work as fast as the demand. Now all of this would seem to indicate that a change in the methods of producing this class of work was in process, and may account for the seeming lack of demand for prosthetic art on the part of the better class of practitioners. We have thought that the commercial laboratories were only patronized by the indolent or incapable practitioners, but we are assured that many of the best practitioners are habitual patrons of them.

If the commercial laboratory is to handle to a large extent our prosthetic art in the future, we can not but entertain serious apprehensions of its degradation to a condition of inefficiency more deplorable than desirable, when the welfare of our patients is considered. It seems incredible that this work is to be put on the "hand me down" basis,—as that is what the method logically implies. It is true that the work produced by these laboratories, from the mechanical standpoint, is many times, high grade and not to be criticized, perhaps, and the fees charged are such that no busy dentist can afford to use his time in such work; but we must not lose sight of the one important fact, that we are professionally bound to supply our patients with the most efficient substitutes possible for their lost dental organs. Now, the question confronting the conscientious practitioner is whether he can better serve his patients through the commercial laboratory, or, by personal attention. We can not see how it is practicable to send an impression, however perfect, to a commercial laboratory and get by return mail a completed denture that has any individuality about it, which we all know is the essential thing in prosthetic art. If it was a fact that all the workmen in these commercial laboratories were expert mechanics the mechanical results might be creditable, but we know that very few men so employed can be classed as even good mechanics. Boys and old worn out dentists, tramps, incapables, and men who have had no professional education or training are too often found in such laboratories undertaking to do work that should require intelligent knowledge of the individual requirements in each case.

The method is surely all wrong; but so long as men will not equip themselves to do prosthetic work in a manner to serve their patients adequately, they will take no pride or even a casual interest in the work, and the commercial laboratory will thrive, because it makes its appeal on a commercial basis to a class of practitioners who lack sufficient discrimination or conscience to resist such appeals.

We do not wish it to be thought that these laboratories do not have a place of useful service, as there is much of this kind of work that can be so handled acceptably. Our complaint is that the profession is pauperizing itself in technical training and failing in its true ministry when it entrusts any considerable part of such service to a mere machine.

We are glad to know that the better men in the profession do not consider the prosthetic side of their practice *infra dig*, but are striving to serve their patients in this department with equal efficiency We would also call attention to the fact that prosthetic art in the past two years has made commendable progress, as may be seen from a review of the literature of our profession. We are printing in this issue extracts from some of the more recent contributions of value, all of which indicate that we are giving more attention to the proper occlusion of artificial teeth, in order that they may render a more adequate service in the present propaganda for better health, as a result of better mastication of our food. Our space is too limited for a larger presentation, but we hope to present some other phases of the subject in subsequent issues.

